# False Negative Twenty‐Nine Montreal Cognitive Assessment Score in Moderately ADL‐Impaired UDS Patients: A Case Series Study of 23 Patients

**DOI:** 10.1002/alz70857_102683

**Published:** 2025-12-24

**Authors:** Youssef A. Ismail, Huda A. Auf, Shahd A. Sadik, Nada M. Ahmed, Yasmeen A Ali

**Affiliations:** ^1^ Faculty of Medicine Port Said Univeristy, Egypt, Port Said, Port Said, Egypt

## Abstract

**Background:**

The Montreal Cognitive Assessment (MoCA) is the most frequently used screening tool to assess the mild cognitive impairment and identify whether specialist required for treatment or not. This case series aimed to investigate the validity of MoCA for recommending or excluding diagnosis of cognitive impairment in patients with moderately‐impaired activities of daily life (ADL).

**Methods:**

This study retrospectively examined the medical records of patients with an optimal cut‐off value of normal cognition (NC), which is 29/30, and also examined the clinician assessment of cognitive impairment symptoms.

**Results:**

Among 87 patients with 29/30 MoCA, 26.4% (23/87) met the diagnostic criteria of dementia. 18 patients were male (represent 78.33% of included study population). All patients experienced meaningful cognitive and executive function impairment. Major impairments were noticeable in memory of 65.20%, orientation of 30.40%, language of 34.80%, visuospatial function of 21.70%, and attention or concentration (10 of 21 patients, 47.62%). The onset of cognitive decline ranged from 41 to 80 years of age, with a median age of 58.5 years. 6 out of 17 patients had presumptive etiologic diagnosis of Alzheimer's Dementia (AD). Moreover, 11 patients were diagnosed with sleep apnea, 2 with hyposomnia/insomnia, 1 with night terrors, and 1 with hypersomnia. 14 patients had 0.5 on Clinical Dementia Scale (CDS), and 9 patients had 1.0. None of the patients had elevated ß‐amyloid, tauopathy or any brain lesions, except only 4 patients had minimal hippocampal atrophy. None of the patients also had any neurological disorders, Diabetes Mellitus (DM), or depression. Only 11 out of 23 patients (47.82%) were able to live independently, and the others either require assistance in complex (39.13%) or basic (8.7%) activities or even completely dependent (4.34%).

**Conclusion:**

MoCA had failed to detect cognitive impairment in patients with moderate ADL‐impairment, even when laboratory and radiological examinations do not indicate typical AD pathology. This study indicates low specificity and negative predictive value (NPV) of MoCA and the necessity for additional screening tools to accurately assess cognitive function.

